# Deciphering Natural Killer Cell Homeostasis

**DOI:** 10.3389/fimmu.2020.00812

**Published:** 2020-05-12

**Authors:** Aline Pfefferle, Benedikt Jacobs, Alvaro Haroun-Izquierdo, Lise Kveberg, Ebba Sohlberg, Karl-Johan Malmberg

**Affiliations:** ^1^Center for Infectious Medicine, Department of Medicine Huddinge, Karolinska Institutet, Stockholm, Sweden; ^2^Department of Cancer Immunology, Institute for Cancer Research, Oslo University Hospital, Oslo, Norway; ^3^The KG Jebsen Center for Cancer Immunotherapy, Institute of Clinical Medicine, University of Oslo, Oslo, Norway

**Keywords:** natural killer cells (NK cells), IL-15, mTOR, homeostasis, NK cell differentiation

## Abstract

Natural killer (NK) cells have a central role within the innate immune system, eliminating virally infected, foreign and transformed cells through their natural cytotoxic capacity. Release of their cytotoxic granules is tightly controlled through the balance of a large repertoire of inhibitory and activating receptors, and it is the unique combination of these receptors expressed by individual cells that confers immense diversity both in phenotype and functionality. The diverse, yet unique, NK cell repertoire within an individual is surprisingly stable over time considering the constant renewal of these cells at steady state. Here we give an overview of NK cell differentiation and discuss metabolic requirements, intra-lineage plasticity and transcriptional reprogramming during IL-15-driven homeostatic proliferation. New insights into the regulation of NK cell differentiation and homeostasis could pave the way for the successful implementation of NK cell-based immunotherapy against cancer.

## NK Cell Development

Natural killer (NK) cells are granular lymphocytes able to unleash stored cytotoxic potential to kill foreign, transformed or infected cells. Compared to other cytotoxic cells, NK cells are not restricted by the need for prior sensitization and can further orchestrate the early phase of the adaptive immune response. NK cells are found in significant numbers in blood, bone marrow, liver, lymphoid organs, lung, and uterus (1) and develop from common lymphoid progenitors in the bone marrow (2). Identification of NK cell precursors outside the bone marrow, namely fetal thymocytes (CD34^+^CD3^−^CD4^−^CD8^−^) and fetal liver cells (CD34^+^CD38^+^) suggests that NK development is not restricted to the bone marrow (3–5). Commitment to the NK cell lineage requires the transcription factors ID2 and E4BP4 along with IL-15 signaling (6–11). The search for an NK-restricted precursor identified CD34^+^CD38^+^CD45RA^+^CD7^+^CD10^+^CD123^−^CD127^−^ cells which can give rise to T-bet^+^ and Eomes^+^ NK cells, two transcription factors central for NK cell maturation in mice (12, 13). Expression of T-bet and Eomes induces CD122 (encoded by *IL2RB*) expression on NK cells, a component of both the IL-2 and IL-15 receptor allowing for survival and effector function signaling to occur (12, 14). Although NK cells belong to the innate immune system, many aspects of T cell biology share a striking similarity with NK cells (15).

## NK Cell Differentiation and Functional Specialization

In humans, NK cells are characterized as CD56^+^CD3^−^ cells. They can be broadly divided into CD56^bright^ and CD56^dim^ subpopulations based on clear functional and phenotypic differences (16–18). CD56^bright^ NK cells are highly responsive to cytokine priming and fulfill an immunomodulatory role. Expression of CCR7, CD62L, CXCR3, CCR5, CCR2, and CXCR4 allows CD56^bright^ cells to home to secondary lymphoid tissues, the liver, skin and bone marrow, where they represent the dominant NK cell subset (1, 19–22). Conversely, cytotoxic CD56^dim^ NK cells, which prioritize activating and inhibitory receptor input, mainly express CX3CR1 and CXCR1 (22), and account for the majority of peripheral blood NK cells (23).

CD56^bright^ NK cells have been suggested as precursors of CD56^dim^ NK cells based on combinatorial approaches including transcriptional studies (24–27). CD56^bright^ NK cells can acquire CD16 expression, effectively transitioning into CD56^dim^ NK cells (18) and CD16^+^CD56^bright^ NK cells exist as functional intermediates (28). Furthermore, CD56^bright^ NK cells are the first lymphocyte population to reconstitute after stem cell transplantation, with CD16 acquisition, decreased surface expression of CD56 and cytotoxic effector functions following at a later time point (29–31). Conversely, in response to cytokine stimulation CD56^dim^ NK cells can adopt a “bright-like” phenotype via upregulation of CD56 (32). CD56^bright^ NK cells also have longer telomers compared to CD56^dim^ NK cells, evidence for having undergone fewer cell divisions (18), and have an increased proliferative capacity compared to CD56^dim^ NK cells (33).

**Figure 1:**
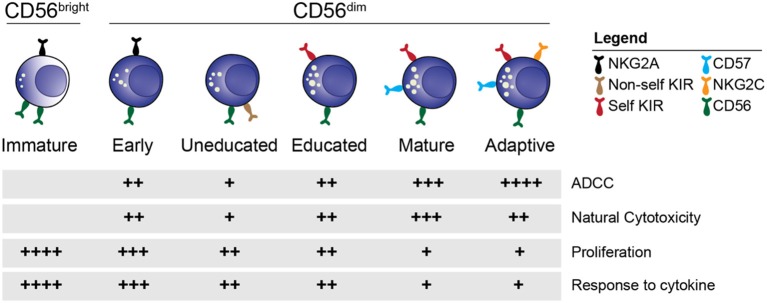
NK cell subsets. Overview of the distinct stages of NK cell differentiation based on phenotypic and functional properties.

Within the CD56^dim^ NK cell population, further distinctions of individual subsets based on phenotypic and functional characteristics can be made (Figure 1) (34). Cells expressing the inhibitory receptor NKG2A are found on the immature end of the spectrum, whereas acquisition of killer cell immunoglobulin like receptors (KIR) gives rise to more differentiated educated and uneducated NK cells with varying functional potential (35). The inhibitory signal strength between self-MHC and NKG2A and KIR fine-tunes the functional potential in a process termed education (35, 36). Expression of CD57, a carbohydrate epitope of unknown binding, is associated with terminal maturation, reduced proliferative capacity, and increased functional potential (37). At the mature end of the spectrum is a unique group of NK cells termed adaptive or memory-like NK cells (38, 39) that can be found in approximately 40% of cytomegalovirus (CMV) seropositive individuals. Adaptive NK cells are characterized by single self-KIR expression, epigenetic downregulation of intracellular signaling molecules and expression of the activating receptor NKG2C and CD57 (40–45). Functionally, adaptive NK cells exhibit increased ADCC activity compared to their non-adaptive counterpart. Although the combination of NKG2A, KIR and CD57 expression is commonly used to define NK cell subsets in humans, this is a simplified model considering that up to 100,000 unique subsets exist within healthy individuals (46).

### Transcriptional Regulation of Human NK Cell Differentiation

Recently, several studies have shed light on the transcriptional regulation of NK cell differentiation. Mouse studies identified the importance of T-bet and Eomes in the differentiation step from immature CD27^+^CD11b^−^ to mature CD27^−^CD11b^+^ NK cells^22^, as well as the role of ZBTB32, IRF2, and IKZF3 in NK cell differentiation (47–49). Bulk sequencing, combined with ChIP sequencing, of human CD56^bright^ and CD56^dim^ NK cells identified the TCF1-LEF-MYC axis within the CD56^bright^ population and the PRDM1-MAF-ZEB2 axis within CD56^dim^ NK cells (50). These transcription factor controlled regulatory schemes within effector cells (CD56^dim^ NK cells) and proliferative precursor cells (CD56^bright^ NK cells) dictated their functional programs as well as localization and trafficking. Expression of BACH2 in CD56^bright^ NK cells repressed BLIMP1 expression while ZEB2 expression in CD56^dim^ NK cells repressed TCF1 expression. The first single-cell RNA sequencing (scRNA seq) study in human NK cells was focused on characterizing the heterogeneity within peripheral blood and organs in both mice and humans (51), without detailing the gene regulatory circuit involved in NK cell differentiation. A recent study from our group (52) set out to delineate the temporal transcriptional regulation of human NK cell differentiation with the aid of scRNA seq. Confirming previous phenotypic and functional studies, we identified two main transcriptional islands, which corresponded to the CD56^bright^ and CD56^dim^ NK cell populations. Intriguingly, they were connected by a narrow bridge which, based on RNA velocity analysis (53), identified a transition from the CD56^bright^ to CD56^dim^ island. This gradual transition between the two main subsets was further corroborated by mapping a confined set of gene trends along pseudotime using Palantir (54).

### Formation of the Functional Template for Education

NK cell education is the process whereby NK cells are functionally tuned via inhibitory interactions mediated between self-MHC and KIR or NKG2A. This is further fine-tuned by the signal strength determined by the number of inhibitory interactions (35, 36). As NK cells do not undergo positive or negative selection, it was initially assumed that they would express a minimum of one inhibitory receptor in order to maintain tolerance to self (55). However, the presence of NKG2A^−^KIR^−^ cells and evidence of completely stochastic KIR repertoires in the developing immune system (56–59) suggested that alternative mechanisms are in play to ensure tolerance to self. Indeed, NK cells that lack self-specific inhibitory receptors circulate in a hypo-responsive state (56, 60, 61). Furthermore, NK cells have the ability to undergo re-education after transfer from one MHC class I environment to another, further validating the need for sustained inhibitory interactions in order to retain functionality (62, 63).

Despite education being a dynamic process that forms an important cornerstone in NK cell functionality, the intracellular mechanism underlying education remained elusive until recently. Multiple models have been proposed, including the arming, the disarming and the rheostat model without a general consensus being reached (35, 64, 65). Discriminating between educated and uneducated NK cells required a functional readout or sequencing of the HLA genes, as no phenotypic readout existed. Recent work from our lab identified granzyme B retention as a sensitive and specific phenotypic readout for education in resting NK cells, putting the core cytolytic machinery itself in the spotlight in the search for an underlying mechanism behind NK cell education (66). Transcriptionally, educated NK cells were identical to uneducated NK cells, but accumulated granzyme B in dense-core secretory lysosomes located close to the centrosome. After target cell interaction, these large granules containing granzyme B were released, in line with increased cytotoxicity compared to uneducated cells lacking these particular granules. Pharmacological inhibition of the protein kinase PIKfyve and genetic silencing of its downstream target, the lysosome-specific calcium channel TRPML1, suggested a model where unopposed activating receptor input leads to remodeling of the lysosomal compartment and loss of dense-core secretory lysosomes in cells that lack self-specific receptors. Downstream of such morphological changes, signaling from acidic calcium stores may fine-tune the cell's functional potential through inter-organelle communication with the endoplasmic reticulum.

Our recent scRNA-seq study (52) identified a gradual increase in expression of effector molecules and genes involved in lysosomal function within the CD56^dim^ population. Furthermore, genes important for vesicle formation and trafficking, such as *RAB27A*, were higher expressed within the CD56^dim^ NK cell subset. Mutations in *RAB27A* cause Griscelli syndrome type 2, resulting in a degranulation defect (67), as Rab27a is recruited to the lytic granules by LFA-1 stimulation, aiding the granule in docking to the plasma membrane (68, 69). Hence, CD56^dim^ NK cells are poised for modulation of the lysosomal compartment mediated via inhibitory and activating receptor input received at the cell surface, resulting in fine tuning of their functionality.

## NK Cell Homeostasis

IL-15 is the main cytokine required for NK cell development, but also for survival, proliferation, metabolism and functionality (70). The importance of IL-15 signaling in NK cell development is best observed through mutations in the receptor components and downstream signaling molecules which, together, present as immunodeficiencies characterized by a lack of NK cells (71–74). Immune cells, including DCs, monocytes and other non-hematopoietic cells trans-present IL-15 on the IL-15Rα chain, which binds to the heterodimer consisting of IL-2Rβ (CD122) and the common γ-chain (CD132) found on the NK cell surface. Downstream signaling is mediated via JAK1/3, allowing for recruitment and activation of the transcription factor STAT5, a survival signal for NK cells (73). A downstream target of STAT5 is the cytokine induced SH2-containing protein (CIS, encoded by *CISH*), which functions as a negative feedback loop by inhibiting the upstream JAK1 (75). *Cish*^−/−^ knockout mice presented increased anti-tumor activity and proliferative capacity as a result of being hyper-responsive to IL-15 signaling (75). Mathematical modeling has been implemented in an attempt to better understand the impact of IL-15 receptor signaling on proliferation. The model predicted that increasing IL-15Rα expression on the cell surface will accelerate the formation of IL-15/IL-15R complexes, particularly at low IL-15 concentrations, until a saturation level is reached and no further proliferative response can be achieved (76). These results highlight the broad and central role for IL-15 in NK cell development, differentiation, homeostasis and priming of effector function.

Quorum sensing, which is a form of chemical communication in bacteria whereby sensing of an autoinducer is used to synchronize group behavior, has recently been proposed to also control immune cell homeostasis (77). For example, colony stimulating factor 1 (CSF1) produced by the surrounding stromal cells is proposed to function as the autoinducer in macrophages, whereby uptake of CSF1 controls the rate of proliferation and survival to maintain a steady population density at homeostasis (78). In T cells, IL-2 replaces CSF1 as the autoinducer, which together with IL-6 has been suggested to also modulate the differentiation from an effector T cell to a central memory T cell (79, 80). The logical autoinducer counterpart in NK cells is IL-15. The threshold for IL-15 induced proliferation is subset-dependent, as observed by the onset of proliferation across the maturation spectrum. This is in line with the concept of quorum sensing, whereby the level of IL-15 in the microenvironment dictates the degree of proliferation and overall size of the population.

### The IL-15 mTOR Axis

The unique role of IL-15 in NK cell biology is largely attributed to the IL-15 mammalian target of rapamycin (mTOR) signaling axis and the metabolic regulation of NK cells. Mouse studies identified a dose-dependent downstream signaling pathway, where high dose IL-15 activated the mammalian target of rapamycin (mTOR) as well as STAT5. mTOR, a serine/threonine kinase consisting of the two complexes mTORC1 and mTORC2, is a master regulator in cells. mTORC1 senses for nutrients in the microenvironment to control metabolism while mTORC2 is involved in controlling the cytoskeletal organization of the cell (81–83). Metabolic reprogramming due to environmental cues has been identified as a key regulatory mechanism behind immune cell differentiation and function in NK cells and other immune cells (81–85). In mice, increased cytokine priming led to metabolic reprogramming, with increased metabolic activity, and a switch in energy source from oxidative phosphorylation (OXPHOS) to glycolysis. An increase in metabolism allowed for IFNγ and granzyme B production, conferring increased functionality which could be reversed through the use of rapamycin, an mTOR inhibitor (81). Viral infection can also activate mTOR leading to metabolic reprogramming, as observed in murine CMV infection^122^. It is possible that in a tumor setting, a lack of available glucose due to high glycolytic activity by the tumor cells could lead to functional inhibition due to lack of mTOR activation (81, 86).

In addition to mediating NK cell functionality via modulation of the cellular metabolism, mTOR may serve as a functional rheostat during NK cell education (82, 87). Educated NK cells exhibited higher basal mTOR activity, which was further increased upon activating receptor ligation and also correlated with the number of inhibitory receptors expressed (87). Expression of SHP-1, a phosphatase required to convert inhibitory receptor input into functional responsiveness, was required for increased mTOR activity in educated cells (88). Conversely, continuous activating receptor input in the absence of inhibitor input dampened mTOR activity. Although education is not transcriptionally regulated in human NK cells, mTOR activity is dependent on its localization to the lysosomal compartment which in turn can be negatively regulated by TRPML1 (89, 90). The connection between lysosomal remodeling during education and metabolic regulation through mTOR is an unexplored area in NK cell biology that warrants further investigation.

### NK Cell Repertoire Dynamics and Intra-Lineage Plasticity

At the donor level, the NK cell repertoire is vastly diverse and unique (46). However, once the NK cell repertoire has been fully formed and in some cases further shaped by pathogens such as CMV, it is well-maintained over time considering the rather rapid turnover of the cells (44, 91). Proliferation therefore plays an important role in replenishing the NK cell pool at steady-state and in maintaining a stable repertoire. NK cell proliferation has mainly been examined in viral or disease settings, where it is associated with rapid cell turnover resulting in subset skewing toward immature NK cells with higher proliferative potential (92–94). In a recent study we asked the question of whether or not stable NK cell repertoires are maintained under homeostatic proliferation (95). We hypothesized that the observed stability was either the result of self-renewal from an immature pool of progenitor cells followed by differentiation, or the result of intra-lineage plasticity (BOX 1). This process has been observed in other immune cells (96, 97) but NK cell plasticity has largely remained unexplored (98).

Box 1 **Cellular plasticity**

Plasticity refers to phenotypic and functional changes occurring within populations of cells. Intra-lineage plasticity, also known as functional plasticity, refers to cells of a given lineage adapting to their surroundings in response to cytokine or receptor input which is translated into transcriptional changes resulting in an altered phenotype and modified functionality. An example of this is macrophages transition between an M1 and M2 phenotype, T cells transitioning from Th to Treg phenotype or ILC subsets transitioning between ILC1- 3 phenotypes.

We developed a simplified model that induced a linear onset of IL-15 induced proliferation with maximal retention of NK cell subsets (based on NKG2A, KIR and CD57) to mimic homeostatic conditions. We observed subset-specific proliferation kinetics, which correlated with mTOR activation. IL-15-induced mTORC1 upregulation prior to proliferation onset could predict downstream proliferation 3 days later at both the donor and subset level. Repeated sampling of the same blood donors over time confirmed stable NK cell repertoires, but also an intrinsic metabolic set point determining the level of mTOR activation in response to IL-15 stimulation.

Despite subset-specific proliferation kinetics, the actual subset frequencies at the population level remained largely stable, suggesting that the repertoires were maintained through intra-lineage plasticity. Indeed, sorting individual NK cell subsets prior to proliferation revealed a surprising degree of cellular plasticity in both immature and mature subsets. Acquisition of NKG2A in sorted KIR+ NK cells was associated with increased proliferative potential and decreased functionality, while the reverse was true for CD57 acquisition by the same subset. Surprisingly, a fraction of CD57^+^ NK cells lost CD57 expression, acquired NKG2A and started to proliferate, suggesting they may not be terminally differentiated. Rapidly cycling educated NK cells underwent transcriptional reprogramming, resulting in a more immature signature, while slowly cycling educated NK cells acquired a more mature signature when compared with baseline subsets.

**Figure 2:**
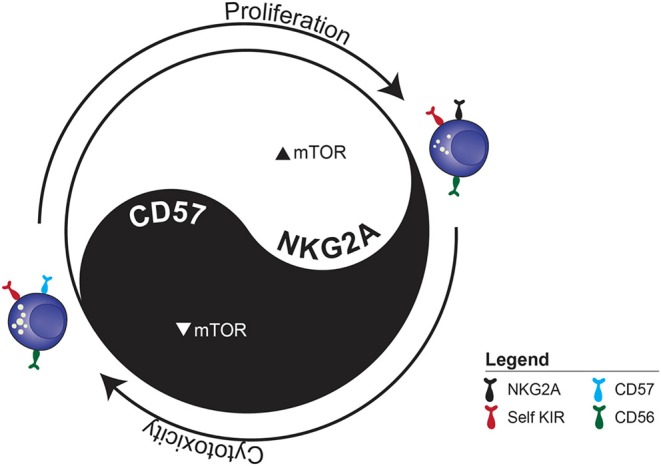
The functional dichotomy between proliferation and cytotoxicity observed during IL-15-induced homeostatic proliferation. The example illustrates the two distinct fates of sorted CD56^dim^ KIR^+^ NKG2A^−^CD57^−^ NK cell depending on whether they acquire NKG2A or CD57.

Our simplified *in vitro* homeostatic NK cell proliferation model allowed us to examine the central role IL-15 plays in maintaining NK cell homeostasis (Figure 2). CD57 expression was associated with a negative influence on mTOR activation and proliferation but enhanced functional potential. Although it is used as a main marker for subset discrimination in NK cells, the function of CD57 remains unknown (37, 99). In neural cells, CD57 has mainly been associated with adhesion proteins, while binding to the IL-6 receptor has also been proposed (100). It would be interesting to further delineate whether CD57 plays a functional role, or if it is simply a surrogate marker for other ongoing cellular modifications.

Due to their differential mTOR activation profile, it is tempting to speculate that NKG2A^+^ and CD57^+^ cells display distinct metabolic profiles. Metabolic reprogramming is responsible for the differentiation of naïve T cells into active effector and later into memory T cells (101–106). The transition of naïve into effector T cells depends on the upregulation of glycolysis and the TCA cycle to provide material for *de-novo* synthesis of proteins, nucleic acids and lipids, whereas formation of memory T cells rely on OXPHOS and fatty acid oxidation (FAO) (107). Such differential use of metabolic programs has also been observed in Th cell subsets (108). In addition, T cell memory formation is influenced through the reorganization of mitochondrial content (109). Interestingly, survival of memory-like NK cells in mice upon CMV infection is dependent on sufficient degradation of dysfunctional mitochondria via mitophagy upon virus clearance (110).

Differences in terms of proliferation speed, phenotype, and functionality between homeostatic and spontaneous proliferation have been investigated in murine T cells (111–113). Spontaneous proliferation, occurring in severely lymphopenic mice, was characterized by a rapid onset of cell division that was cytokine-independent. Homeostatic proliferation, on the other hand, occurred in mildly lymphopenic mice at a slower division rate and required both cytokine and T cell receptor (TCR) stimulation. The proliferation-induced phenotype was reverted after removal of the proliferation cues and cytotoxic capacity of CD8^+^ T cells was lost during the initial phase of intense proliferation (111–113).

Considering the asymmetric PI3K and mTOR activity post-cell division observed in T cells and its role in controlling differentiation fate and the functional dichotomy in proliferating vs. arrested NK cells (101–106), it would be of interest to do microscopy studies of cellular division or functional interactions with target cells. Based on the induced transcriptional signature in rapidly cycling NK cells, which included both RNA-modifying metabolic genes and actin filament organization genes (95), the loss of functionality in rapidly cycling cells may be due to underlying deficits at the immune synapse. Conjugate formation experiments combined with F-actin staining at the site of the immune synapse would further shed light on the loss of functionality observed during intense homeostatic proliferation.

### NK Cell Homeostasis *in vivo*

Given the essential role of IL-15 on NK cells, stimulation of IL-15 signaling pathways has been explored in clinical settings (114–120). In this regard, three main strategies have been pursued; using recombinant-human IL-15 (116) generated by *E. coli*, an IL-15 superagonist, ALT-803 (114, 115) and transfection of an IL-15 containing CAR construct (121, 122). These have been thus far tested in Phase I and II clinical trials, with recombinant-human IL-15 and ALT-803 both showing moderate success in inducing NK cell proliferation and activation *in vivo* and in particular cases inducing disease remission. A limitation of this approach has been the induction of some minor side effects relating to an increased inflammatory environment. Subcutaneous delivery of the compounds has resulted in a partial reduction of these side effects (115). Recent pre-clinical studies have highlighted the potential of combination therapy using this IL-15 signaling stimulation and other immunotherapeutic agents such as monoclonal antibodies or check-point blockade (118, 119). Reflecting this, there are currently more than 100 registered clinical trials exploring IL-15 stimulation via either of these two methods in a series of different cancer settings (www.clinicaltrials.gov). *In vitro*, transfection with an IL-15 containing CAR construct sustained autonomous NK cell growth over 42 days and increased systemic IL-15 serum levels were observed in mouse studies (121). However, in 11 patients treated in a Phase I/II trial, the detection of infused CAR^+^ NK cells by flow cytometry was limited to the first 2 weeks post infusion (122).

In the setting of stem cell transplantation, NK cells are the first lymphocyte population that can be detected following engraftment (123). Their ability to mediate graft-vs.-leukemia (GVL) effects is vital for elimination of residual disease, as increased number of NK cells after transplantation result in better treatment outcome (124, 125). Insights into the specificity of NK cell alloreactivity, determined by specific combinations of KIR and HLA, paved the way for the ground-breaking discovery of a potential role of NK cells in mediating GVL in haploidentical HSCT against AML (126, 127). Studies aiming at harnessing NK cell alloreactivity in the context of HSCT have recently been reviewed (128, 129). The indication that NK cells may deliver a potent GVL effect in the setting of HSCT inspired the whole NK cell community to develop adoptive NK cell therapy based on transfer of “KIR ligand mismatched” NK cells across HLA barriers to promote missing self-recognition. Whereas many studies did not find a beneficial effect of genetic KIR ligand mismatch, calculation of the functional dose of KIR ligand mismatched NK cells was associated with less relapse after NK cell therapy against AML (130–132).

A recent series of Phase III clinical trials have brought mTOR inhibition to the forefront of transplantation (117, 120). In both of these studies a series of patients received Sirolimus, an alternate name for rapamycin, as a prophylactic against *graft vs. host disease* (GVHD). Sandmaier et al. reported that inclusion of Sirolimus to the standard calcineurin inhibitor treatment showed decreased incidence of grade 2–4 acute GVHD. Similarly, increased overall survival, and progression-free survival, as well as decreased non-relapse related mortality was observed in the sirolimus treated group. Due to the clear improved benefit of Sirolimus treatment, the trial carried out by Sandmaier et al. was terminated prior to complete patient recruitment.

On the other hand, a parallel study by Gooptu et al. did not report significant differences compared to standard treatment regarding GVHD incidence, progression free survival nor overall survival (117). This discrepancy may be due to the differences in standard treatment and dosage of Sirolimus between the two studies. In the latter study, the immune reconstitution was evaluated at a series of timepoints up to 24 months. Sirolimus treatment led to a decreased lymphocyte cell count in the first 3 months of treatment, and an increased ratio of regulatory T cells to CD8^+^ T cells throughout the first 6 months of treatment. Lower NK cell counts were observed in the first month following Sirolimus treatment, although this recovered to similar levels compared to standard treatment by the 3rd month. Given the phenotypic and functional heterogeneity of NK cell subsets and the critical role of mTOR and IL-15 signaling in driving NK cell plasticity, it would be of great interest to further evaluate the precise composition of the NK cell compartment during such therapies.

### Cytokine-Based Expansion Protocols for NK Cell Therapy

There are several up to date and comprehensive reviews describing the prospects of using various preparations of NK cells in cell-based immunotherapy (133, 134). These include strategies based on autologous and allogeneic NK cells that have been stimulated by various cytokines alone or in combination with irradiated feeder cells expressing membrane bound cytokines such as IL-21 or IL-15 (121, 135–138). Therefore, we will focus on a general discussion on how these protocols may drive dramatic phenotypic and functional changes to the NK cell repertoire (34, 95). To expand large numbers of cells for multi-dosing schemes, many strategies are based on supra-physiological levels of cytokines, including any combination of IL-2, IL-15, IL-12, and IL-18 (139, 140). However, this can result in severe and acute cytokine deprivation post-infusion as the cells become “addicted” to cytokines (BOX 2). Severe side-effects (141, 142) prevent patients from being treated with the same cytokines and persistence is further limited through clearance of infused NK cells by host immunity.

Box 2 **Cellular addiction**

Cytokine priming results in intracellular signaling changes occurring within cells. Continuous stimulation with non-physiological cytokine levels can result in an altered cellular state, which requires further cytokine stimulation to support survival. This can be referred to as cytokine-dependence or addiction, whereby cytokine withdrawal can lead to detrimental consequences to the cell.

### The Balance Between Pro- and Anti-apoptotic Molecules During IL-15 Driven Proliferation

We recently set out to characterize the mechanism behind IL-15 addiction and withdrawal in expanded NK cells. NK cells exhibited a dose-dependent IL-15 addiction, where high-doses induced rapid proliferation, skewing toward a naïve phenotype, and subsequent crash upon cytokine withdrawal (143). Timing of IL-15 dosing is crucial for NK cell survival and effector function as chronic high-dose IL-15 stimulation leads to decreased viability of NK cells with reduced respiratory spare capacity and functional activity (144).

Numerous pro- and anti-apoptotic genes make up the apoptosis network balancing the outcome of the cell during various types of stimulations (70, 145–147). Within resting NK cells, BCL-2 has been identified as an important anti-apoptotic protein which can be further upregulated through IL-15 stimulation, leading to downstream STAT5, but not mTOR activation (82, 148). In actively proliferating NK cells, MCL-1 expression is vital to maintain viability (149). BIM is a pro-apoptotic molecule and its downstream target BAX is directly inhibited by BCL-2 (147). In murine effector CD8^+^ T cells, increased BIM levels are balanced by increased BCL-2 levels, expression of which dictates the amount of BIM that can be tolerated (150). Similarly, in murine NK cells, the BCL-2/BIM ratio was influenced by IL-15 stimulation and withdrawal, whereby changes in the ratio could render the cells sensitive to cell death (70, 150, 151). In line with these studies, we observed an IL-15 dose-dependent increase in BCL-2, MCL-1, and BIM expression. BCL-2 and MCL-1 were both crucial for survival in NK cells stimulated with high-dose IL-15 as shown through blocking experiments. Interestingly, rapidly cycling NK cells exhibited reduced BCL-2 levels compared to slowly or non-cycling NK cells during their expansion phase, in line with T cell proliferation studies (145).

**Figure 3:**
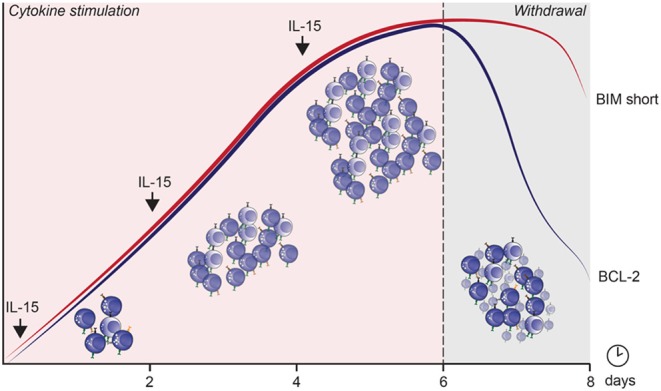
The mechanism by which apoptosis is induced in cycling NK cells after IL-15 induced cytokine dependence and subsequent withdrawal. The curves represent expression of BIM short (red) and BCL-2 (blue) over culture time.

After cytokine withdrawal, anti-apoptotic proteins decreased, and a potent apoptosis-inducing splice variant, BIM S, (152, 153) was preferentially upregulated in proliferating cells and remained highly expressed until 24 h after cytokine withdrawal. This severely altered the pro/anti-apoptotic ratio, exposing rapidly cycling cells to high levels of toxic BIM S within 24 h after cytokine withdrawal (Figure 3). The importance of these apoptotic proteins in IL-15 mediated survival and function has also been observed in murine studies (70). How homeostatic and induced proliferation affects NK cell cytotoxicity, and how apoptosis is induced in cycling cells upon cytokine withdrawal, has potentially important implications for current cell therapy expansion protocols.

## Concluding Remarks

NK cells circulate in a pre-primed state full of effector molecules, such as granzyme B and perforin, and have a natural ability to kill cancer cells. Based on their cytotoxic capacity they hold great potential in the clinic as a cancer treatment, made evident by the number of ongoing clinical trials. However, to date most completed and ongoing clinical trials are based on the transfer of cytokine-activated polyclonal NK cell populations from donors with very variable NK cell repertoires. To fully harness the clinical potential of NK cells, future trials need to be founded on recent breakthroughs in our understanding of the vast repertoire diversity and the fundamental mechanisms that govern the intrinsic functional potential of distinct NK cell subsets at steady state and following cytokine stimulation.

Understanding how NK cells repertoires are formed and maintained over time, and what functional roles individual cell subsets perform in a homeostatic setting, are important to improve current therapies and develop future treatment strategies. Generating an “ideal” NK cell product for treatment could involve modifying existing cells to improve functionality, expanding highly cytotoxic subsets while ensuring retention of functionality or designing a “synthetic” genetically engineered killer cell from induced pluripotent stem cells.

Furthermore, we need to understand how NK cells are functionally shaped by their surroundings. The soluble factors, metabolic cues, fluctuations in oxygen levels, and pH encountered by an NK cell in the tumor microenvironment are very different from steady state and their impact on NK cell function and persistence cannot be underestimated. This is particularly difficult to study in the human setting, with mouse models only providing an approximation.

By understanding the basic biology, from development to differentiation to receptor and cytokine input, we will build up our tool kit that can then be applied to design and develop effective treatment strategies. After all, the “natural” killing capacity is there, we just need to understand how to harness it.

## Author Contributions

AP made the figures. AP and K-JM wrote the paper. LK, AH-I, BJ and ES contributed to the writing of the paper.

## Conflict of Interest

K-JM is a consultant at Fate Therapeutics. The remaining authors declare that the research was conducted in the absence of any commercial or financial relationships that could be construed as a potential conflict of interest.
